# General self-efficacy, not musculoskeletal health, was associated with social isolation and loneliness in older adults during the COVID-19 pandemic: findings from the Hertfordshire Cohort Study

**DOI:** 10.1007/s40520-023-02676-5

**Published:** 2024-02-03

**Authors:** Gregorio Bevilacqua, Leo D. Westbury, Ilse Bloom, Jean Zhang, Wendy T. Lawrence, Mary E. Barker, Kate A. Ward, Elaine M. Dennison

**Affiliations:** 1 Medical Research Council (MRC) Lifecourse Epidemiology Centre, University of Southampton, Southampton General Hospital, Tremona Road, Southampton, SO16 6YD UK; 2 National Institute for Health and Care Research (NIHR), Southampton Biomedical Research Centre, University of Southampton and University Hospital Southampton National Health Service (NHS) Foundation Trust, Southampton, UK; 3 School of Health Sciences, Faculty of Environmental and Life Sciences, University of Southampton, Southampton, UK; 4 MRC Unit The Gambia, London School of Hygiene and Tropical Medicine, Banjul, The Gambia; 5 School of Biological Sciences, Victoria University of Wellington, Wellington, New Zealand

**Keywords:** Social isolation, Loneliness, Self-efficacy, Ageing, COVID-19 pandemic, Musculoskeletal conditions

## Abstract

**Background:**

Social isolation and loneliness are prevalent among older adults. This study investigated factors influencing worsening social isolation and loneliness in community-dwelling older adults during the COVID-19 pandemic, focusing on musculoskeletal conditions, falls, and fractures.

**Methods:**

We studied 153 participants from the Hertfordshire Cohort Study. Baseline assessments (2019–20) included osteoporosis, clinical osteoarthritis, fractures after age 45 years, falls in previous year, and lifestyle factors. Self-efficacy was assessed using a shortened General Self-Efficacy Scale. Social isolation was assessed using the 6-item Lubben Social Network Scale. Follow-up (2020–21) assessments included social isolation and loneliness using the 6-item De Jong-Gierveld scale for emotional, social, and overall loneliness.

**Results:**

Baseline median age was 83.1 years. A history of smoking predicted worsening social isolation (*p = *0.046). Being married (*p = *0.026) and higher self-efficacy scores (*p = *0.03) predicted reduced social isolation at follow-up. Greater alcohol consumption was associated with higher overall loneliness (*p = *0.026). Being married was related to a 36% (95% CI: 3%, 58%) reduction in emotional loneliness (*p = *0.037). No musculoskeletal condition was associated with social isolation or loneliness. However, we observed a 22% (14%, 30%; *p < *0.001) reduction in emotional loneliness and a 12% (4%, 20%; *p = *0.003) reduction in overall loneliness per unit increase in self-efficacy score.

**Conclusions:**

No musculoskeletal condition was associated with increased social isolation or loneliness, but longitudinal studies in larger samples are required. Greater self-efficacy was associated with reduced social isolation and reduced loneliness. Interventions promoting self-efficacy in older adults may reduce isolation and loneliness in this age group.

## Introduction

Meaningful social relationships are fundamental for individuals’ lives and health, influencing their physical and psychological wellbeing [1]. Social isolation and loneliness are common issues among older adults worldwide. A recent meta-analysis by Teo and colleagues reported a global prevalence of social isolation, defined as the objective lack of social contact and interactions with friends, family, and the community [2], of 26% among community-dwelling older adults [3]. Loneliness, which is the subjective experience of dissatisfaction with the quantity and/or quality of one’s social relationships [4, 5], is reported by 18% to 27% of older adults in the UK alone [6, 7]. Both social isolation and loneliness are growing public health concerns: social isolation has been associated with both physical and psychological adverse health outcomes, such as poor physical capability, myocardial infarction, stroke, depression and mortality [8–14]. Similarly, loneliness has been linked to higher mortality rates, as well as poorer cardiovascular and mental health [11, 12, 15]. In fact, the risks associated with loneliness have been reported to be comparable to those associated with major health risk factors such as obesity and smoking [9].

Following the World Health Organization’s recognition of the coronavirus disease (COVID-19), caused by the severe acute respiratory syndrome coronavirus 2 (SARS-CoV-2), as a pandemic, the United Kingdom entered its first national lockdown on March 23rd 2020, and people were required to stay at home as much as possible and only socialise with members of their own household [16]. Specifically, individuals throughout the UK were allowed to leave their homes exclusively for the following purposes: shopping for basic necessities (i.e., food and medicines); performing physical exercise alone or with another member of the same household; seeking medical care or providing medical care for a vulnerable person; travelling to and from work, if remote working was not possible [16]. These restrictions remained in place until June 2020, when non-essential retail re-opened. However, despite further and gradual easing of lockdown rules, shielding and social distancing were still recommended for the most vulnerable until September 2021 [17].

While the strategy of social distancing and self-isolation has been successful in reducing infections, hospitalizations, and deaths due to COVID-19, it has also had negative consequences: older adults, who are at a higher risk of adverse health outcomes after contracting the virus, experienced increased social isolation and loneliness due to these policies [18–21], with potentially severe longer term consequences for both physical and mental health.

Poor musculoskeletal health might be expected to have an impact on social isolation as individuals with severe osteoarthritis or recurrent falls might spend less time away from their own homes as their mobility and confidence might be impacted.

Using data from a longitudinal cohort of English community-dwelling older adults, we considered what factors were associated with social isolation and loneliness, and with longitudinal changes in social isolation during the COVID-19 pandemic. In particular, we looked at potential relationships with osteoarthritis, osteoporosis, falls, and fractures as exposures: we hypothesised that poor musculoskeletal health may be related to increased social isolation and loneliness during the pandemic.

## Methods

### The Hertfordshire Cohort Study

Participants were recruited from the Hertfordshire Cohort Study (HCS), a population-based sample of men and women born between 1931 and 1939 in Hertfordshire and originally recruited to study the relationship between growth in infancy and the subsequent risk of adult diseases [22].

### Baseline stage for the current analysis (November 2019–March 2020)

Between November 2019 and March 2020, 176 participants from the HCS (94 men and 82 women) were visited at home by a trained fieldworker who administered a questionnaire that included information on medical history, medication use, lifestyle and social isolation. The visits also included measurements of height and weight to calculate body mass index (BMI), and the performance of the Short Physical Performance Battery (SPPB) tests [23].

### Follow-up stage for the current analysis (November 2020–June 2021)

Between November 2020 and June 2021, 153/176 of these participants (82 men and 71 women) agreed to complete a postal follow-up questionnaire which included validated tools to assess social isolation and loneliness. Previously, between July 2020 and February 2021, 125/176 participants (65 men and 60 women) were contacted by telephone and consented to complete a questionnaire administered by a trained researcher; this also included questions to measure social isolation and loneliness. When data on social isolation and loneliness were not available from the follow-up postal questionnaire, we used those recorded during the telephone survey; no other information from this telephone survey was used in this study.

### Ascertainment of social isolation and loneliness

Social isolation was assessed at both timepoints using the 6-item Lubben Social Network Scale (LSNS-6), which has been validated to assess social networks and social support and to screen for social isolation in older people [24]. The LSNS-6 tool measures the number and frequency of social interactions with friends (three items) and family members (three items). Each answer is assigned a score ranging from 0 (“none”) to five (“nine or more”), and the overall final score ranges from 0 (indicating high isolation or few social resources) to 30 (indicating low isolation or many social resources). Social isolation was defined as a LSNS-6 score < 12, in accordance with Lubben et al. [24]. The LSNS-6 has been shown to have good internal consistency across samples of community-dwelling older adults [24–26].

Loneliness was assessed at follow-up using the shortened 6-item De Jong-Gierveld scale, which has been proved to be a reliable and valid measuring tool for overall, emotional and social loneliness [27]. Social loneliness can be described as an individual’s perception that the number of their relationships is smaller than is considered desirable, while emotional loneliness can be defined as an individual’s perception of a lack of intimacy in confidant relationships [28]. The De Jong-Gierveld scale consists of two 3-item subscales for emotional loneliness and social loneliness. The overall loneliness score ranges from 0–6, with higher scores indicating greater general loneliness. The subscales have a score of 0–3, with higher scores indicating greater emotional or social loneliness.

### Independent variables

Participants’ height and weight were measured at baseline and used to calculate body mass index (BMI). Smoker status was dichotomized into ‘never smoked’ and ‘ever-smoked’, depending on the participants’ answers to the questions ‘Do you currently smoke?’ and ‘Have you ever been a smoker?’. Participants were asked how often they drink different types of alcohol (beer, wine, spirits, etc.) and how much they normally drink each time. This was used to estimate their alcohol consumption in units per week.

Physical activity was assessed via the Longitudinal Aging Study Amsterdam Physical Activity Questionnaire (LAPAQ) which has been validated for use in older populations [29].

Physical performance was assessed using the Short Physical Performance Battery (SPPB) tests: gait speed, ability to perform chair rises, and balance testing [23]. The SPPB has good to excellent reliability and good reproducibility [30]. A physical capability score was derived from the tests in accordance with the SPPB scoring guidelines [23]. The scoring scale ranged from a minimum of 0, indicating no capability, to a maximum of 12, representing optimal physical capability.

Marital status was dichotomized into ‘currently married’ and ‘not currently married’, based on the categories provided by participants (i.e., single, married, divorced or separated, and widowed).

General self-efficacy was assessed using a shortened version of the Generalised Self-Efficacy Scale (GSE), developed by Schwarzer and Jerusalem in 1981, and has been shown to have good psychometric properties [31, 32]. This scale measures an individual’s self-belief in coping with the demands, tasks and challenges of life in general. The shortened GSE consists of the following five items: “I can always manage to solve difficult problems if I try hard enough”; “I can find a way to get what I want even if someone is trying to stop me”; “It is easy for me to stick to my aims and reach my goals”; “I am calm when things are difficult because I know I can cope”; and “If I am in trouble I can usually find a way out”. Answers to each item range from ‘strongly disagree’ (to which the lowest value of 1 is assigned, indicating low efficacy) to ‘strongly agree’ (to which the highest value of 4 is assigned, indicating high efficacy). For the current study, we treated the GSE score as an untransformed continuous variable.

Participants were asked whether a doctor had ever diagnosed them with any of the following comorbidities: high blood pressure, diabetes, lung disease (e.g., asthma, chronic bronchitis, emphysema or COPD), rheumatoid arthritis, multiple sclerosis, Parkinson’s disease, peripheral arterial disease, thyroid disease, vitiligo, depression, heart disease (e.g., heart attack, angina or heart failure), stroke and cancer. Number of comorbidities was calculated to obtain a marker of morbidity burden.

The presence of osteoporosis was assessed by asking the question ‘Have you been told by a doctor that you have osteoporosis?’.

Clinical osteoarthritis and fracture history were obtained from previous visits of the cohort. Clinical osteoarthritis was ascertained during a previous pass the of the HCS (2011) by physical examination of the hand, knees, and hips performed by a trained fieldworker. Clinical OA was defined based on algorithms developed by the American College of Rheumatology [33]. Fracture history since age 45 years was ascertained from a previous 2017 pass of the HCS via a fieldworker-administered questionnaire. In addition, in 2019–2020 (timepoint 1), participants were asked to report any fracture they experienced in the previous 12 months. Self-reported fractures were thus ascertained by combining answers from both questionnaires.

### Statistical methods

Participant characteristics were described using summary statistics. Sex differences in participant characteristics were assessed using t-tests, Wilcoxon rank-sum tests, chi-squared tests, and Fisher's exact tests as appropriate. The following characteristics at baseline were regarded as exposures: age, BMI, smoking status, alcohol consumption, physical activity, physical performance score, marital status, GSE score, number of comorbidities, diagnosis of osteoporosis, OA (knee hip, or hand), fracture since age 45 years, and a fall in the previous year. Linear regression was used to examine exposures in relation to LSNS-6 score at follow-up (after adjustment for age, sex and follow-up time) and in relation to annualised change in LSNS-6 score from baseline to follow-up (after adjustment for age, sex and baseline LSNS-6 score). Finally, exposures were examined in relation to emotional, social and overall loneliness scores at follow-up using Poisson regression with robust variance estimation; age, sex and follow-up time were included as adjustments.

Men and women were pooled to increase statistical power. Analyses were performed using Stata, release 17.0; *p < *0.05 was regarded as statistically significant. The analysis sample comprised 153 participants with data on at least one of the follow-up outcomes.

## Results

### Participant characteristics

Characteristics of the participants included in the analysis sample are presented in Table 1. Median (lower quartile, upper quartile) age was 83.1 (81.5, 85.3) years. At baseline, 10 (13.3%) men and 11 (17.5%) women were socially isolated (LSNS-6 score < 12); corresponding figures among men and women at follow-up were 14 (18.7%) and 8 (12.7%), respectively. Median (lower quartile, upper quartile) loneliness scores at follow-up were as follows: emotional (men: 0.0 (0.0, 1.0), women 1.0 (0.0, 1.0)); social (men 1.0 (0.0, 2.0), women 0.0 (0.0, 2.0)); and overall (men 2.0 (0.0, 3.0), women 1.0 (0.0, 3.0)). Differences in the distribution of these social isolation and loneliness characteristics between men and women were not statistically significant (*p > *0.19 for all associations).

**Table 1:** Participant characteristics of the analysis sample

Participant characteristic	Mean (SD); median (lower quartile, upper quartile); *N* (%)		*p* value for sex difference	Missing values
	Men (*n = *82)	Women (*n = *71)		
***Ascertained at baseline (2019–2020)***				
Age (years)	82.8 (81.5, 84.9)	83.4 (81.5, 85.4)	0.485	0
BMI (kg/m^2^)	27.6 (3.4)	26.9 (4.5)	0.302	3
Ever smoked	39 (47.6%)	21 (30.0%)	**0.027**	1
Alcohol (units per week)	3.3 (0.3, 10.1)	1.5 (0.0, 6.6)	**0.010**	0
Physical activity in mins/day (LAPAQ)	110.7 (64.3, 152.1)	152.9 (102.9, 231.4)	** < 0.001**	3
Physical performance score (SPPB)	9.0 (7.0, 11.0)	8.0 (6.0, 9.0)	**0.024**	6
Currently married	61 (74.4%)	35 (49.3%)	**0.001**	0
General self-efficacy score	14.9 (1.9)	15.0 (2.0)	0.763	1
Number of comorbidities	2.0 (1.0, 2.0)	2.0 (1.0, 2.0)	0.806	0
Osteoporosis	7 (8.5%)	20 (28.2%)	**0.001**	0
Clinical knee, hip or hand OA*	12 (16.0%)	23 (32.9%)	**0.018**	8
Fracture since age 45 years**	12 (17.4%)	22 (36.1%)	**0.016**	23
Fall in previous year	30 (36.6%)	15 (21.1%)	**0.036**	0
LSNS-6 score	17.3 (5.5)	17.3 (5.3)	0.968	15
Socially isolated (LSNS-6 < 12)	10 (13.3%)	11 (17.5%)	0.501	15
***Ascertained at follow-up (2020–2021)***				
LSNS-6 score	16.8 (5.8)	17.1 (4.9)	0.788	15
Socially isolated (LSNS-6 < 12)	14 (18.7%)	8 (12.7%)	0.340	15
Emotional loneliness score	0.0 (0.0, 1.0)	1.0 (0.0, 1.0)	0.199	15
Social loneliness score	1.0 (0.0, 2.0)	0.0 (0.0, 2.0)	0.458	7
Overall loneliness score	2.0 (0.0, 3.0)	1.0 (0.0, 3.0)	0.407	19

SD: Standard deviation, LAPAQ: Longitudinal Aging Study Amsterdam Physical Activity Questionnaire, SPPB: Short physical performance battery, LSNS-6: Lubben Social Network Scale (6-item), OA: Osteoarthritis

*Clinical OA was ascertained in 2011

**Fractures since age 45 years were ascertained in 2017 and at baseline (2019–2020)

### Baseline characteristics in relation to LSNS-6 score at follow-up and annual change in LSNS-6 score from baseline to follow-up

Results from this analysis are presented in Table 2. Significant baseline correlates of higher LSNS-6 score at follow-up were being currently married (difference in LSNS-6 score at follow-up compared to those who were not currently married: 2.26 [95% CI: 0.28, 4.23], *p = *0.026) and higher GSE score (0.52 (0.05,0.99) per unit increase in GSE score, *p = *0.030). Ever smoking was the only baseline characteristic associated with change in LSNS-6 score from baseline to follow-up; on average, ever smokers experienced greater declines in LSNS-6 scores compared to never smokers (*p = *0.046). Baseline physical activity time, physical performance measures, number of comorbidities, osteoporosis, osteoarthritis, fracture since age 45 years, and falls in the previous year were not associated with either LSNS-6 score at follow-up or annual change in LSNS-6 score.

**Table 2:** Baseline characteristics in relation to LSNS-6 score at follow-up and annual change in LSNS-6 score from baseline to follow-up

Baseline characteristic	LSNS-6 at follow-up (adjusted for age, sex and follow-up time)	P-value	Annual change in LSNS-6 (adjusted for age, sex and baseline LSNS-6)	*p* value
	Estimate (95% CI)		Estimate (95% CI)	
Age (years)	− 0.25 (− 0.63,0.13)	0.195	− 0.26 (− 0.58,0.06)	0.108
BMI (kg/m^2^)	0.02 (− 0.21,0.25)	0.864	− 0.02 (− 0.22,0.17)	0.809
Ever smoked	− 0.66 (− 2.56,1.23)	0.490	**− 1.60 (− 3.17,− 0.03)**	**0.046**
Alcohol (units per week)	0.03 (− 0.06,0.11)	0.539	0.00 (− 0.08,0.07)	0.896
Physical activity (mins/day)	0.00 (− 0.01,0.01)	0.510	0.00 (− 0.01,0.01)	0.511
Physical performance score (SPPB)	0.28 (− 0.09,0.65)	0.139	0.23 (− 0.08,0.54)	0.152
Currently married	**2.26 (0.28,4.23)**	**0.026**	0.44 (− 1.29,2.16)	0.619
General self-efficacy score	**0.52 (0.05,0.99)**	**0.030**	0.24 (− 0.16,0.64)	0.235
Number of comorbidities	− 0.67 (− 1.50,0.17)	0.115	− 0.17 (− 0.88,0.55)	0.642
Osteoporosis	− 1.17 (− 3.74,1.40)	0.369	− 0.57 (− 2.70,1.57)	0.599
Clinical knee, hip or hand OA*	− 1.26 (− 3.61,1.10)	0.292	− 0.91 (− 2.65,0.83)	0.301
Fracture since age 45 years**	0.06 (− 2.16,2.28)	0.959	0.27 (− 1.73,2.27)	0.789
Fall in previous year	0.76 (− 1.25,2.77)	0.456	0.39 (− 1.31,2.08)	0.654

Estimates correspond to the difference in outcomes per unit increase in the baseline characteristic or the presence versus absence of the baseline characteristic. Statistically significant (*p < *0.05) associations are highlighted in bold

SPPB: Short physical performance battery, LSNS-6: Lubben Social Network Scale (6-item), OA: Osteoarthritis

*Clinical OA was ascertained in 2011

** Fractures since age 45 years were ascertained in 2017 and at baseline (2019–2020)

### Baseline characteristics in relation to loneliness scores at follow-up

Associations between baseline characteristics and emotional, social and overall loneliness scores at follow-up are presented in Table 3. Higher alcohol consumption was related to higher overall loneliness (*p = *0.026). Being currently married was related to a 36% (95% CI: 3%, 58%) reduction in emotional loneliness score (*p = *0.037). Per unit increase in GSE score was related to a 22% (14%, 30%) reduction in emotional loneliness score (*p < *0.001) and a 12% (4%, 20%) reduction in overall loneliness score (*p = *0.003). These associations were examined after adjustment for sex, age and follow-up time. Baseline physical activity time, physical performance, number of comorbidities, osteoporosis, osteoarthritis, fracture since age 45 years, and falls in the previous year were not associated with any of the loneliness measures at follow-up.

**Table 3:** Baseline characteristics in relation to loneliness scores at follow-up (adjusted for age, sex and follow-up time)

Baseline characteristic	Emotional loneliness		Social loneliness		Overall loneliness	
	Estimate (95% CI)	*p* value	Estimate (95% CI)	*p* value	Estimate (95% CI)	*p* value
Age (years)	1.05 (0.98,1.13)	0.155	0.98 (0.90,1.05)	0.527	1.01 (0.96,1.07)	0.715
BMI (kg/m^2^)	1.01 (0.96,1.07)	0.603	1.00 (0.95,1.05)	0.892	1.00 (0.96,1.04)	0.857
Ever smoked	1.05 (0.67,1.65)	0.819	0.95 (0.66,1.38)	0.800	1.07 (0.78,1.45)	0.687
Alcohol (units per week)	1.02 (1.00,1.04)	0.084	1.01 (0.99,1.02)	0.430	**1.01 (1.00,1.02)**	**0.026**
Physical activity (mins/day)	1.00 (1.00,1.00)	0.882	1.00 (1.00,1.00)	0.395	1.00 (1.00,1.00)	0.424
Physical performance score (SPPB)	1.00 (0.92,1.09)	0.946	0.99 (0.92,1.07)	0.841	1.01 (0.95,1.07)	0.826
Currently married	**0.64 (0.42,0.97)**	**0.037**	0.88 (0.60,1.30)	0.530	0.81 (0.60,1.09)	0.165
General self-efficacy score	**0.78 (0.70,0.86)**	** < 0.001**	0.93 (0.82,1.04)	0.209	**0.88 (0.80,0.96)**	**0.003**
Number of comorbidities	1.07 (0.88,1.30)	0.493	0.99 (0.83,1.18)	0.927	1.02 (0.91,1.14)	0.736
Osteoporosis	0.90 (0.50,1.61)	0.718	1.48 (0.99,2.23)	0.058	1.16 (0.81,1.65)	0.412
Clinical knee, hip or hand OA*	0.87 (0.53,1.44)	0.594	1.00 (0.62,1.59)	0.987	0.94 (0.64,1.39)	0.760
Fracture since age 45 years**	0.89 (0.51,1.55)	0.676	0.98 (0.61,1.57)	0.928	0.95 (0.64,1.43)	0.823
Fall in previous year	0.93 (0.56,1.53)	0.770	0.89 (0.62,1.29)	0.552	0.92 (0.67,1.27)	0.612

Estimates correspond to the multiplicative increase in outcomes per unit increase in the baseline characteristic or the presence versus absence of the baseline characteristic; an estimate of 1.2 would reflect a 20% increase and an estimate of 0.8 would reflect a 20% decrease. Statistically significant (*p < *0.05) associations are highlighted in bold

SD: Standard deviation, LAPAQ: Longitudinal Aging Study Amsterdam Physical Activity Questionnaire, SPPB: Short physical performance battery, LSNS-6: Lubben Social Network Scale (6-item), OA: Osteoarthritis

*Clinical OA was ascertained in 2011

**Fractures since age 45 years were ascertained in 2017 and at baseline (2019–2020)

## Discussion

In a population of UK community-dwelling older adults, we found that the prevalence of social isolation at baseline was generally in line with previous estimates for social isolation among older adults, ranging between 15 and 40% [34]. The prevalence of social isolation in our population sample as a whole was approximately 16% at both timepoints; this was lower than the 19% prevalence of social isolation reported in UK participants with a mean (SD) age of 70.3 (16.8) years from the English Longitudinal Study of Ageing (ELSA) [35]. In addition, other studies found an increase in social isolation among older adults during the COVID-19 pandemic [19, 20]; in our study, mean LSNS-6 scores were lower at follow-up compared to baseline, suggesting that participants were generally more socially isolated during the COVID-19 pandemic. Since no information about how our participants perceived the impact of the pandemic and its associated restrictions on their lives was available for this sample, it remains difficult to ascertain whether these factors had a direct influence on social isolation and loneliness within our sample. However, a small subgroup of 12 individuals from this same sample was interviewed over the phone between March and October 2020 to collect qualitative data and explore how older adults’ experiences and behaviours changed over time throughout the first wave of the pandemic [36]. During these semi-structured discussions, participants emphasized that, due to the pandemic and its related restrictions, they experienced issues with shopping and food accessibility (e.g., not getting the types of food they wanted and not being able to access specific shops), limitations on activities (e.g., unavailability of cardiac rehabilitation classes or walking groups), disruptions to healthcare (e.g., cancelled hospital appointments or community prevention groups), and feelings of isolation and loneliness [36]. In addition, the timing of our quantitative data collection coincided with the pandemic to such an extent that it is difficult to believe that the observed changes occurred independently of such context.

We hypothesized that a medical history of osteoporosis, OA, or previous fractures and falls would be associated with risk of isolation and loneliness in this population. Reassuringly, we found that none of these factors were associated with worsening social isolation and loneliness after one year of follow-up despite high prevalence of these musculoskeletal conditions in this population. To the best of our knowledge, no previous study has addressed the potential impact of musculoskeletal health on isolation and loneliness in older adults: previous literature seems to have focused on relationships going in the opposite direction in different age groups. For instance, Christiansen and colleagues found that loneliness, but not social isolation, was associated with increased odds of osteoarthritis among Danish adolescents and young adults [37]. It is not immediately clear why, in our sample, musculoskeletal health was not associated with social isolation and loneliness after one year of follow-up. It is possible that our finding is due to the unusual context of the COVID-19 pandemic, during which individuals could not or would not engage in social activities as much as they did in pre-pandemic times, regardless of their musculoskeletal health. This finding warrants further investigation in larger cohorts.

In our study, we found that being currently married and having higher GSE scores at baseline were both associated with higher LSNS-6 scores at follow-up, which are suggestive of being less socially isolated. Being an ever-smoker (current or previous smoker) at baseline, on the other hand, was associated with greater decline in LSNS-6 score, which indicates worsening isolation. Similarly, being married at baseline was associated with a reduction in follow-up emotional loneliness, while higher GSE scores were associated with a reduction in both emotional and overall loneliness. Conversely, higher alcohol consumption at baseline was found to be associated with higher overall loneliness at follow-up.

The finding that being married provided protection against social isolation and loneliness in our sample is not unexpected. In comparison to married participants, individuals without a partner were more likely to be living alone. This becomes especially significant when considering the circumstances of the first national UK lockdown, where socialising was restricted to individuals within the same household. Therefore, those who were not cohabitating with others were potentially exposed to a higher risk of experiencing both social isolation and loneliness. There are limited data from other studies, but interestingly, a recent study by Murayama and colleagues, conducted in the first year of the pandemic with over 50,000 Japanese men and women aged 15–79 years, found that married and cohabiting participants were more likely to be socially isolated than their counterparts [19]. However, these diametrically opposed findings might be ascribed to the wide age range used in the Japanese study and to cultural differences between the UK and Japan. It is possible that in our population sample, having a partner may have supported and facilitated interactions outside the household, in contrast to Japanese couples who tended to confine their social interactions within the household. On the other hand, Murayama and colleagues also reported higher levels of loneliness among unmarried participants [19], in line with our own finding.

We found that baseline higher GSE scores were associated with lower social isolation, and lower emotional and overall loneliness at follow-up. It is plausible that being confident about one’s ability to cope with the demands, tasks and challenges of life facilitates social engagement. A recent study conducted among 150 US community-dwelling older adults aged 65 years and older found that coping self-efficacy was associated with decreased odds of loneliness [38]. Other studies have also reported that greater general self-efficacy is associated with decreased loneliness [39–41].

To the best of our knowledge, our observation that baseline ever-smokers experienced worsening social isolation from baseline to follow-up is a novel finding. A previous study be Ikeda and colleagues, conducted in two ageing cohorts from the UK (ELSA) and Japan (Japan Gerontological Evaluation Study), reported that older adults with better social networks were more likely to quit smoking compared to isolated participants [42]. It has also been previously reported that socially isolated individuals are more likely to be smokers [43]. Our study suggests that this association may be bidirectional and calls for further investigation.

Similarly, we reported an association between higher alcohol consumption and greater overall loneliness. Excessive drinking is known to be associated with adverse mental health conditions such as depression and stress [44], and loneliness is positively associated with depression [45], which might explain the association observed in the current study. A study of US young adults found no association between alcohol consumption and loneliness [46]. However, the study population in question was notably younger than our own, and the underlying motivations for their drinking habits may differ considerably. Furthermore, the relationship between alcohol consumption and loneliness has not been thoroughly explored, and thus, it warrants further investigation to gain a comprehensive understanding [46, 47]. Lastly, it is important to note that loneliness data were not available at baseline: it is possible that individuals with higher loneliness at follow-up already had higher loneliness at baseline, rather than alcohol consumption at baseline being casually related to loneliness at follow-up.

Our study has some limitations. First, our population sample may not be entirely representative of the wider UK population of the same age, as all participants were born in the county of Hertfordshire, where they were still living in their homes, and were all Caucasian. Nevertheless, it has been previously demonstrated that the HCS is representative of the general population in terms of anthropometric body build and lifestyle factors (e.g., smoking and alcohol intake) [48], although ‘healthy’ responder and survivor biases are evident within the HCS [49]. In addition, the fairly small sample size prevented us from robustly examining whether correlates of social isolation and loneliness differ between men and women. However, when stratifying analyses by sex, associations were broadly similar between men and women (data not shown). Similarly, the relatively small size of our sample may have limited us in fully examining possible associations of poor musculoskeletal health with risk of isolation and loneliness, so studies in larger longitudinal samples of a wider age range are warranted. Moreover, some variables were self-reported in our study and, therefore, recall bias cannot be ruled out. Lastly, fractures could not be captured for part of the time window between 2017 and 2019–2020 as only fractures over the previous 12 months were asked at the latter time point. On the other hand, our study has a number of strengths: the longitudinal design of our study allows for the associations reported to be regarded as potentially causal; we measured both social isolation and loneliness using validated and reliable tools; and our study sample was recruited from a population of community-dwelling older adults that have been extensively phenotyped and well characterized with regard to lifestyle and past medical history.

## Conclusion

In a sample of UK community-dwelling older adults we found that none of the musculoskeletal conditions examined were associated with worsening social isolation or loneliness during the COVID-19 pandemic, although longitudinal studies in larger population samples are warranted. Being married and having greater self-efficacy were associated with lower social isolation and loneliness after one year of follow-up. These findings are important because of the potentially modifiable nature of self-efficacy. By promoting self-efficacy among older adults, we may have the potential to alleviate the adverse effects of social isolation and loneliness in this population.

## Data Availability

Hertfordshire Cohort Study data are accessible via collaboration. Initial enquires should be made to EMD (Principal Investigator). Potential collaborators will be sent a collaborators’ pack and asked to submit a detailed study proposal to the HCS Steering Group.
